# Genomic Analysis and Taxonomic Characterization of Seven Bacteriophage Genomes Metagenomic-Assembled from the Dishui Lake

**DOI:** 10.3390/v15102038

**Published:** 2023-09-30

**Authors:** Haoyun Cai, Yifan Zhou, Xiefei Li, Tianqi Xu, Yimin Ni, Shuang Wu, Yongxin Yu, Yongjie Wang

**Affiliations:** 1 College of Food Science and Technology, Shanghai Ocean University, Shanghai 201306, China; haoyuntsai@163.com (H.C.); yifan.zhou@pasteur.fr (Y.Z.); felix2015_mail@126.com (X.L.); xutianqi98@163.com (T.X.); nemo.ni@outlook.com (Y.N.); swu@shou.edu.cn (S.W.); yxyu@shou.edu.cn (Y.Y.); 2 Laboratory for Marine Biology and Biotechnology, Qingdao National Laboratory for Marine Science and Technology, Qingdao 266000, China; 3 Laboratory of Quality and Safety Risk Assessment for Aquatic Products on Storage and Preservation, Ministry of Agriculture and Rural Affairs, Shanghai 201306, China

**Keywords:** bacteriophage, genome, taxonomy, freshwater lake, virus diversity

## Abstract

Viruses in aquatic ecosystems exhibit remarkable abundance and diversity. However, scattered studies have been conducted to mine uncultured viruses and identify them taxonomically in lake water. Here, whole genomes (29–173 kbp) of seven uncultured dsDNA bacteriophages were discovered in Dishui Lake, the largest artificial lake in Shanghai. We analyzed their genomic signatures and found a series of viral auxiliary metabolic genes closely associated with protein synthesis and host metabolism. Dishui Lake phages shared more genes with uncultivated environmental viruses than with reference viruses based on the gene-sharing network classification. Phylogeny of proteomes and comparative genomics delineated three new genera within two known viral families of *Kyanoviridae* and *Autographiviridae*, and four new families in *Caudoviricetes* for these seven novel phages. Their potential hosts appeared to be from the dominant bacterial phyla in Dishui Lake. Altogether, our study provides initial insights into the composition and diversity of bacteriophage communities in Dishui Lake, contributing valuable knowledge to the ongoing research on the roles played by viruses in freshwater ecosystems.

## 1. Introduction

Viruses are the most abundant entities and harbor the majority of genetic diversity in aquatic ecosystems by far [1]. It is estimated that there are 10^31^ virus-like particles (VLPs) on Earth [2], playing a crucial role in natural processes by regulating global biogeochemical processes and facilitating microbial evolution through horizontal gene transfer [3]. Typically, phages are responsible for the lysis of about 10–50% of marine bacteria [4].

Freshwater systems house an estimated 1.76 × 10^27^ VLPs [5]. The scientific community has shown increasing interest in exploring viruses in freshwater habitats, even though the majority of viruses remain uncultured. High-throughput sequencing (HTS) has played an irreplaceable role in enabling us to examine and understand viral community characteristics across various freshwater systems without the need for cultivation or reliance on specialized isolation methods [6,7,8,9]. This has led to the discovery of the potential key interactions between viruses and hosts in deep freshwater lakes with metagenomes [10], the known diversity of eukaryotic and prokaryotic viruses in the Yangtze River [11], seasonal alternations in the viral community structure in artificial freshwater reservoir [12], and the various lake viromes worldwide [13,14,15]. Countless genomic fragments were assembled and characterized from viral metagenomic data [16], with the constant development and improvement of computational tools and standards for uncultivated viruses [17,18,19,20,21], allowing for the accurate identification of novel viral groups.

Dishui Lake, situated in Shanghai’s Pudong New Area, is the city’s largest artificial lake. Sourced from the Huangpu River, the lake accepts surface runoff and discharges into the East China Sea, undertaking important functions such as flood control and drainage, and displacing water bodies. The water quality as well as the diversity of bacteria and phytoplankton in Dishui Lake have been investigated and are closely related to human activities [22,23,24,25]. During our metagenomic and metaviromic investigation of eukaryotic microbial viruses in Dishui Lake, diverse large/giant dsDNA viruses and their parasitic viruses of virophages have been discovered, identifying a new tripartite cell–virus–virophage (C-V-v) infection system that consists of green algae, large green algal virus, and virophage [26,27,28,29]. To date, the bacteriophage community in the lake has remained largely unexplored and awaits further research in deciphering their enigmatic characteristics.

In this study, we obtained complete genomes of seven novel phages from Dishui Lake based on metagenomics. A variety of approaches were used to analyze their distinctive genomic features, affiliations, and potential hosts. Three of them were identified to be new members of *Autographviridae* and *Kyanoviridae*, and each of the other four was classified as a new family in *Caudoviricetes*. Collectively, unique bacteriophages were discovered in Dishui Lake for the first time, which provides fresh perspectives on the variety and distribution of phages in offshore freshwater ecosystems as well as contributes to the isolation and cultivation of specific phages from Dishui Lake.

## 2. Materials and Methods

### 2.1. Metagenomic Data Source

The Dishui Lake metagenomic datasets have been previously reported in our work [26,27]. Briefly, surface water samples at a depth of 1.5 m were collected from Dishui Lake, Shanghai (121°55′27″ E, 30°53′55″ N) and filtered through 0.22 µm (pore size) membrane (GSWP, Merck Millipore). The total DNA of microbial biomasses was extracted using QIAamp Fast DNA Stool Mini Kit (QIAgen). 

High-throughput sequencing was performed by the Illumina Miseq sequencing platform at Shanghai Personalbio Technology Co., Ltd. (Shanghai, China). Miseq sequencing library was constructed with insert sizes of approximately 400 bp fragments (the Nextera DNA Flex Library Prep Kit, Illumina), and paired-end sequencing of 2 × 251 bp was carried out. Mate pair (MP) library of approximately 3 kb in length was also prepared and sequenced for validating the correct assembly of sequences. Removing the sequencing adapters and trimming low-quality (<Q20) reads was performed using FastQC and NGS QC Toolkit [30]. The high-quality reads ranging in length from 50 to 250 bp were retained and then assembled into contigs varying from 250 bp to 50 kbp [27].

### 2.2. Viral Genome Assembly

Contigs containing conserved phage genes were considered as the references for assembly with clean sequence dataset (minimum overlap ≥25 bp, minimum overlap identity ≥ 95%) and contigs dataset (minimum overlap ≥ 25 bp, minimum overlap identity ≥ 97%). The completeness and precision of the assembled viral genomes were double-checked by MP library mapping and PCR validation. PCR primers were designed based on the assembled genomic sequences, and DNA templates for PCR were the same DNA samples as used for metagenomic sequencing. PCR amplicons were purified and sequenced. The sequences were aligned with the assembled sequence scaffolds. The Geneious R10.0.9 (https://www.geneious.com accessed on 12 May 2018) and MP library were used to assemble and check the topology of sequences, respectively. Viral contigs (>10 kb) were screened by Cenote-Taker2 [31] to assess Dishui Lake viral diversity.

### 2.3. Genome Characterization

Open reading frames (ORFs) of seven Dishui Lake phage genomes were predicted with Prodigal V2.6.3 in meta mode [32], and their potential functions were annotated through BLASTP [33] search against the NCBI non-redundant (nr) database (http://blast.ncbi.nlm.nih.gov/ accessed on 16 June 2022) and the Batch CD-Search tool of the NCBI Conserved Domains Database (CDD) [34]. HHpred search [35] (https://toolkit.tuebingen.mpg.de/hhpred accessed on 16 June 2022) was conducted against Pfam-A_v35, PDB_mmCIF70_31_Jul, UniProt-SwissPort-viral70_3_Nov_2021, and COG_KOG_v1.0 databases. Only if the protein sequence had a high probability (>95%) was it recognized when compared to the HHpred database. Auxiliary metabolic genes (AMGs) were predicted using VIBRANT v1.2.1 (Virus Identification By iteRative ANnoTation) with default settings [36]. The tRNA genes were identified using the tRNAscan-SE search program [37]. BACPHLIP was employed to determine the phage lifestyle (lysogenic or lytic) based on its genome [38]. Phage genome maps were visualized using CGView [39].

### 2.4. Retrieval of Viruses Related to Dishui Lake Phages in Environmental Datasets

To figure out the affiliations of the Dishui Lake phages with other uncultivated viral genomes (UViGs), the ORFs of seven Dishui Lake phages were queried against the IMG/VR4 database (released on 20 September 2022) [40]. A virus was considered a relative [41,42] if I) it shared at least 50% of proteins (E < 1 × 10^−5^, identity ≥ 30% and coverage ≥ 50%, bitscore ≥ 50) with the query genome in common, and II) it had a genome size neither shorter than 50% nor longer than 150% compared to the respective Dishui Lake phages.

### 2.5. Protein-Cluster-Based Gene-Sharing Network

Seven Dishui Lake phage genomes and the selected environmental UViGs were combined with the vConTACT v2.0 database [43] which was set to ProkaryoticViralRefSeq211-Merged, containing reference sequences of prokaryotic viruses (released on 25 April 2022), with standard parameters (options: --rel-mode Diamond --db ProkaryoticViralRefSeq211-Merged --pcs-mode MCL --vcs-mode ClusterONE --threads 32). Relevant genomes that were clustered with the Dishui Lake phages (similarity score ≥ 1) were chosen for follow-up analysis. The visualization of gene-sharing networks was performed by Cytoscape v3.9.1 (http://cytoscape.org/ accessed on 10 October 2022) [44]. 

### 2.6. Proteomic Trees

Proteomic trees were generated using the Viral Proteomic Tree server (ViPTree) [45], with the whole genomes of phages obtained in this study, known ICTV-ratified families, and the reference sequences of Virus–Host DB [46] to describe their family-level taxonomic status and relationship. The trees were edited with the Interactive Tree of Life (iTOL) online tool [47] (https://itol.embl.de/upload.cgi accessed on 20 April 2023). 

### 2.7. Comparative Genomic Analysis

The proportion of orthologous components (ratio of orthologous gene numbers) between viral genomes was calculated using CompareM (https://github.com/dparks1134/ CompareM; --evalue 1 × 10^−5^ --per_identity 30) (accessed on 18 March 2023). The outcome was transformed into a matrix with the tidyr R package, and heatmaps were generated using the pheatmap R package. Dishui Lake phages and the related viruses shown in the proteomic trees were clustered and calculated by using VIRIDIC [48] according to the nucleotide-based intergenomic similarity thresholds at 95% for species and 70% for genus. BLASTN parameters were set as: ‘-word size 7-reward 2-penalty-3-gapopen 5-gapextend 2′. Global alignment and comparison between genomes in each homologous gene cluster and the gene order were interactively visualized with Clinker [49] (https://github.com/gamcil/clinker accessed on 25 March 2023) with default settings. 

### 2.8. Phylogenetic Analysis

Phylogenetic analyses were conducted by using the protein sequences of phage signature genes of terminase large subunit (TerL) and DNA-dependent RNA polymerase of *Autographiviridae*, identified in Dishui Lake phages and their homologous counterparts detected in other viruses in the NCBI GenBank database (BLASTP; E-value < 1 × 10^−5^; identity > 30%; and alignment coverage > 50%). Protein sequences were aligned with MUSCLE in the MEGA X software [50] under the default settings, and alignments containing more than 50% gaps were excluded. Neighbor-joining trees were reconstructed with 1000 bootstrap value and visualized using iTOL. Fourteen conserved core genes in the family *Kyanoviridae* as well as DSL-LC02 and DSL-LC03 were concatenated, aligned by using MAFFT v1.5 [51], and trimmed with TrimAL v1.4 [52]. Phylogenetic analysis was carried out with FastTree v2.1 [53] under Whelan and Goldman (WAG) substitution mode.

### 2.9. Phage Host Prediction

Host prediction was performed using iPHoP [54], HostG [55], and the CrisprOpenDB tool [56]. Meanwhile, the Dishui Lake phage tRNA genes were used as queries to search the NCBI nr database via BLASTn for identifying potential hosts, and top hits (at least 95% coverage and 95% identity) were deemed authentic matches [57]. 

## 3. Results

### 3.1. Viral Community Composition in Dishui Lake

In total, 415 viral contigs (>10 kb) were primarily identified in the Dishui Lake metagenomic dataset by Cenote-Taker2 (Table S1), and they were assigned to *Kyanoviridae* (14.0%), *Autographiviridae* (2.2%), *Inoviridae* (5.8%), and other virus categories (3.1%). The remaining contigs were grouped into unclassified phages (69.9%) or viruses (5.0%). Overall, *Kyanoviridae* was the most abundant identifiable virus in Dishui Lake.

### 3.2. Genomic Features of Dishui Lake Phages

According to the metagenomic sequence assembly, reference assembly (contig extending), and PCR-based genomic sequence checking, complete genomes of seven phages were discovered in the Dishui Lake metagenome, and their genome size ranged between 29 kbp and 173 kbp (Table 1). For ease of reference, they were named DSL-LC01 (OQ999401), DSL-LC02 (OR032571), DSL-LC03 (OR003938), DSL-LC04 (OR003939), DSL-LC05 (OR003940), DSL-LC06 (OR003941), and DSL-LC07 (OQ999402), respectively.

**Figure 1:**
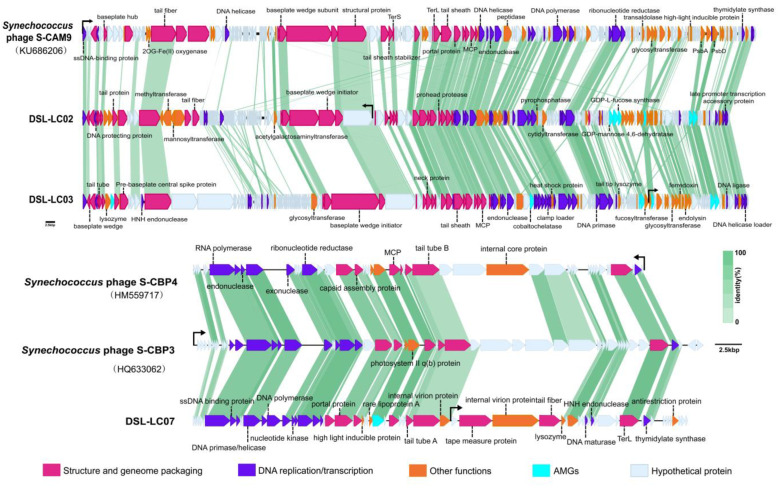
Whole-genome alignments of DSL-LC02, DSL-LC03, and DSL-LC07 with their closest related viruses of *Synechococcus* phage S-CAM9, *Synechococcus* phage S-CBP3, and *Synechococcus* phage S-CBP4. Colored coding sequences represent different functional groups. Green linkages between genomes indicate sequence similarity (>30%) of shared homologous genes.

**Figure 2:**
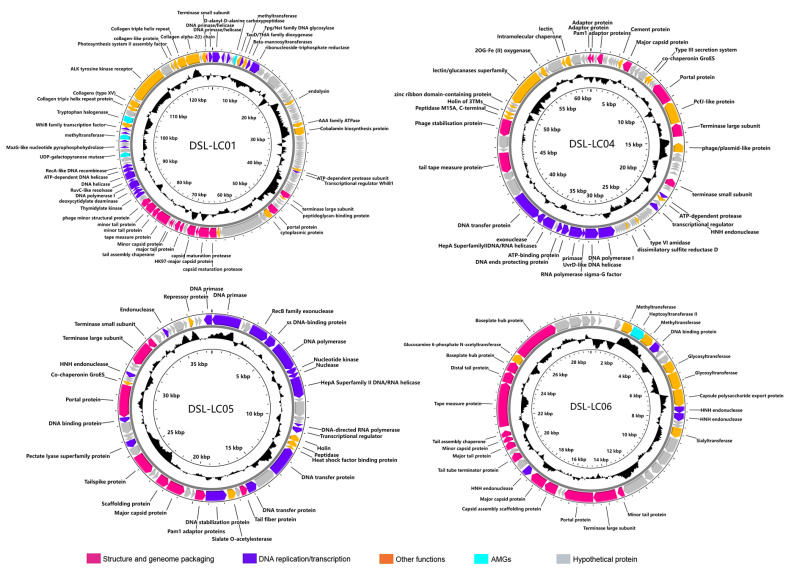
The genome maps of DSL-LC01, DSL-LC04, DSL-LC05, and DSL-LC06. The outside circle represents ORFs. The black peaks denote the (G+C) mol% (outward indicates higher than the whole genome average (G+C) mol%, and inward indicates the opposite). ORFs are colored according to their functional categories.

The combination of the nr database comparison, Batch-CD search for conserved structural domains, and the HHpred search for remote relatives was applied for accurate annotation of viral ORFs (Table S2). A total of 44.8% (377/841) ORFs were functionally annotated, including genes encoding replication proteins present in all genomes, such as DNA primase, helicase, and polymerase (Figure 1 and Figure 2). The predicted structural proteins in Dishui Lake phages were MCP, minor capsid protein (mCP), portal proteins, baseplate hub proteins, the tail tube, and tape measure proteins (Figure 1 and Figure 2). The HK97-fold major capsid proteins (MCPs) are the vital proteins in *Duplodnaviria* [58]. The remaining 464 (55.2%) were predicted to be hypothetical proteins or unknown ORFs (Figure 1 and Figure 2). Additionally, a total of 19 AMGs were detected in the Dishui Lake phage genomes by VIBRANT and are associated with seven distinct metabolic categories (Figure 1 and Figure 2) (Figure S1). The top three are glycan biosynthesis and metabolism, followed by energy metabolism and carbohydrate metabolism. DSL-LC01 appeared to be a lysogenic phage, while the others were lytic ones (Table 1).

### 3.3. Protein-Cluster-Based Gene-Sharing Network

A total of 238 relevant viral sequences of Dishui Lake phages were retrieved from the IMG/VR4 dataset (Table S3). These sequences along with the viral sequences in the NCBI Bacterial and Archaeal Viral RefSeq V211 with ICTV and NCBI taxonomy (released on 25 April 2022) were subjected to the gene-sharing network analyzing with the seven Dishui Lake phages by using vConTACT2 (a total of 4723 input sequences). Notably, ICTV has updated the classification of viruses, eliminating the former order *Caudovirales* and the families *Myoviridae, Siphoviridae,* and *Podoviridae* [21]. Accordingly, the following results were interpreted based on the updated classification information in NCBI and ICTV taxonomy.

**Figure 3:**
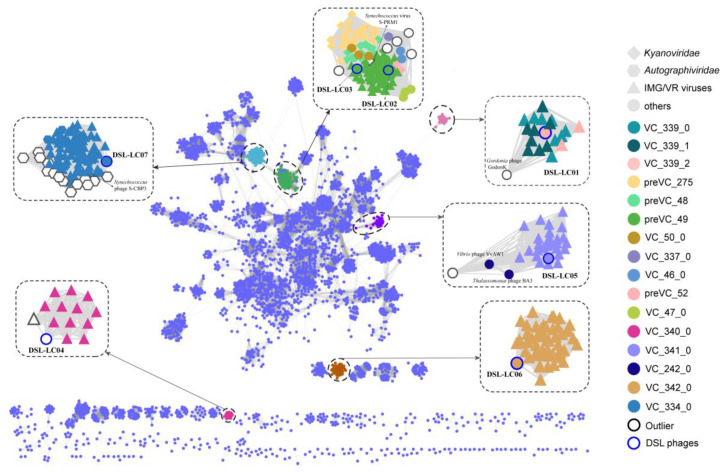
The gene-sharing network of Dishui Lake phages, reference prokaryotic DNA viruses, and related UViGs from the IMG/VR dataset. Each node represents a genome, and edges signify the similarity between genomes based on shared protein clusters (PCs). Dishui Lake phages and the nodes that first neighbor them are shown in different colors, and other viruses are in light blue. The VCs associated with Dishui Lake phages are enlarged.

Six Dishui Lake phages formed five VCs of DSL-LC01 (VC_339_2), DSL-LC02 (VC_49_31), DSL-LC03 (VC_49_32), DSL-LC05 (VC_341_0), DSL-LC06 (VC_342_0), and DSL-LC07 (VC_334_0) (Figure 3 and Table S4). All Dishui Lake phages shared more genes with IMG/VR uncultivated environmental viruses compared with the viruses in the NCBI RefSeq. DSL-LC04 was classified as an outlier to VC_340_0 comprising the UViGs solely. DSL-LC06, together with 50 UViGs, formed VC_342_0, which represents an orphaned group. The grouping of DSL-LC05 resembled that of DSL-LC04 and DSL-LC06 but additionally connected to unclassified *Caudoviricetes* viruses of *Vibrio* phage VvAW1 (NC_020488.1), *Rhodoferax* phage P26218 (NC_029061.1), and *Thalassomonas* phage BA3 (NC_009990.1). DSL-LC01 was in VC_339_2 and connected to the outlier *Gordonia* phage GodonK (NC_048176.1), a member of the floating genus *Godonkavirus* in *Caudoviricetes*. The VC_334_0 where DSL-LC07 was clustered contained 84 viral sequences, 75 of which were UViGs, with 70 and eight classified to *Autographiviridae* in IMG/VR and RefSeq, respectively. This indicates that DSL-LC07 is most likely a member of this viral family. DSL-LC02 and DSL-LC03 were grouped to the same VC. Most of the viruses that were related to them affiliated with the newly established family *Kyanoviridae*, and others belonged to floating genera and unclassified viruses in the class *Caudoviricetes*. In addition to environmental viruses, *Synechococcus* phage S-CAM9 (NC_031922.1) shared the most genes with DSL-LC02 and DSL-LC03 (Figure 1).

### 3.4. Proteomic Trees

Based on the above classification results, the exemplar viruses from *Kyanoviridae* (57 sequences) and *Autographiviridae* (373 sequences) in the ICTV official Master Species List (MSL) were used as reference sequences to determine whether DSL-LC02, DSL-LC03, and DSL-LC07 were members in these families. 

**Figure 4:**
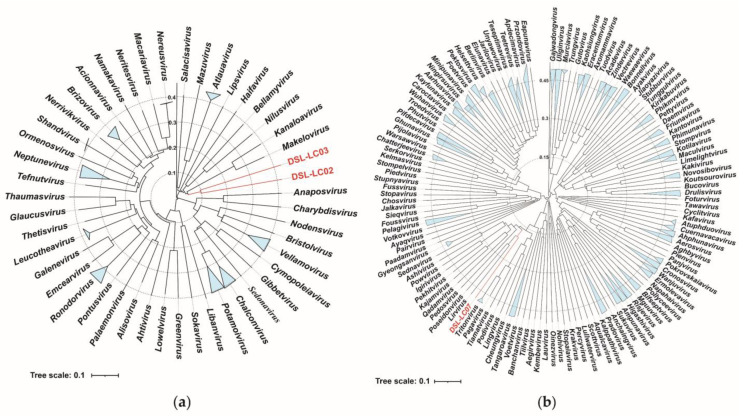
Proteomic trees of (**a**) DSL-LC02, DSL-LC03, and reference viruses in the family *Kyanoviridae*, and (**b**) DSL-LC07 and reference viruses in the family *Autographiviridae*. The Dishui Lake phages and their branches are labeled in red. Genus containing more than one virus was collapsed into blue triangle.

The proteomic trees were reconstructed by using ViPTree based on the whole genomic sequences of DSL-LC02, DSL-LC03, and the *Kyanoviridae* phages. As shown in Figure 4a, DSL-LC02 and DSL-LC03 were grouped together, closely related to two known cyanophages (*Synechococcus* virus S-PRM1of *Makelovirus* and S-CAM9 of *Kanaloavirus*), and were embedded in *Kyanoviridae*. The branching scale between the members of *Neptunevirus* was minimal (around 0.27), while DSL-LC02 and DSL-LC03 had a branching value of about 0.1. Accordingly, the grouping of ViPTree laterally indicates that each of these two DSL phages likely represents a new genus of the family.

DSL-LC07 was clustered with *Synechococcus* phage S-CBP3 of *Lirvirus* and S-CBP4 of *Poseidonvirus*) (Figure 4b). The branching scales between members of the same genus in the *Autographiviridae* showed that *Uliginvirus* had the smallest branching scale of about 0.3. By contrast, the branching scale between DSL-LC07 and known viruses was at about 0.2, which suggests that DSL-LC07 could be classified at the taxonomic rank of new genus in this family.

**Figure 5:**
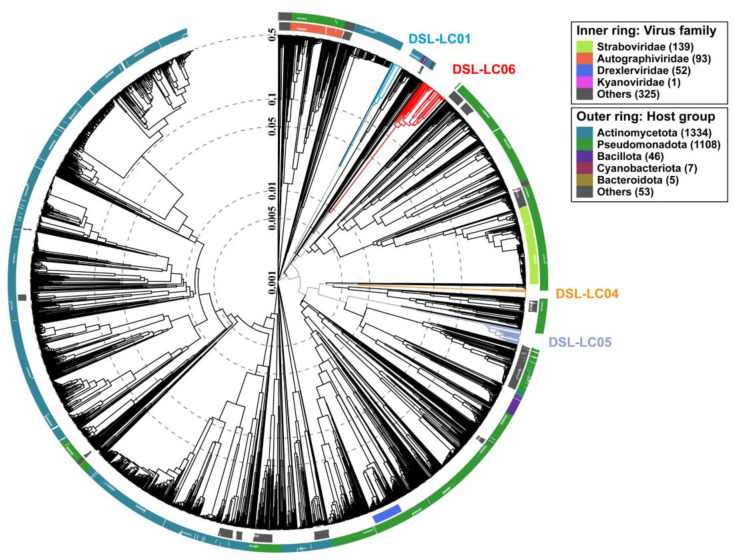
Proteomic tree of DSL-LC01, DSL-LC04, DSL-LC05, DSL-LC06, and reference sequences from the Virus–Host DB. The branches containing Dishui Lake phages and related viruses are indicated in sky blue, red, yellow, and light blue, respectively, and their corresponding enlarged subtrees are shown in Figure S2.

Similar to the above results of the gene-sharing network (Figure 3), Dishui Lake phages of DSL-LC01, DSL-LC04, DSL-LC05, and DSL-LC06 were not closely related to known virus families but to UViGs (Figure 5 and Figure S2). Collectively, the results suggest that these four Dishui Lake phages could be assigned to new families along with closely related UViGs (if complete genome available) of *Caudoviricetes*.

### 3.5. Orthologous Fraction and Nucleotide Identity of Viral Genomes

To further evaluate their classification on either the family or genus level, the orthologous fraction (OF) was calculated for DSL-LC02, DSL-LC03, and DSL-LC07 with CompareM. Notably, Dishui Lake phages of DSL-LC01, DSL-LC04, DSL-LC05, and DSL-LC06 were excluded from such analysis because of the absence of known families associated with them. As a result, DSL-LC02 and DSL-LC03 shared 20–50% of OFs with the representative members in *Kyanoviridae* (Figure S3), and DSL-LC07 shared 18–60% of OFs with *Autographiviridae* viruses. The OFs shared between these Dishui Lake phages and reference viruses are within the range of OFs shared among members of each of these two families (Figure S3). Moreover, the OFs shared between viruses of the same genus were 70–90%. The taxonomical placements of DSL-LC02, DSL-LC03, and DSL-LC07 based on the OFs are in agreement with the above proteomic tree.

**Figure 6:**
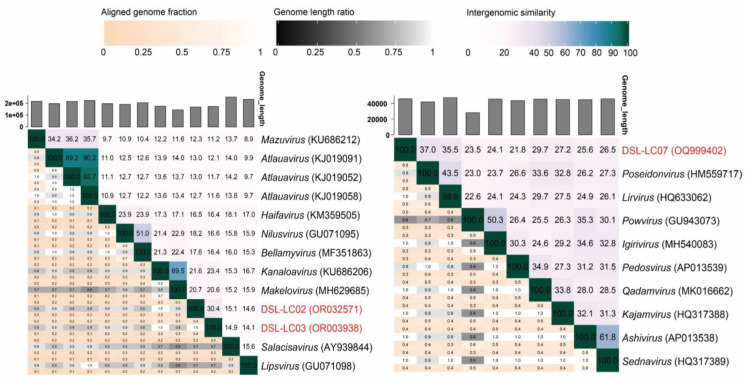
VIRIDIC-generated heatmaps of DSL-LC02, DSL-LC03, DSL-LC07, and their closely related viruses in the proteomic trees (Figure 4). The heatmaps incorporate intergenomic similarity values and alignment metrics of aligned fraction of genomes and genome length ratio. Dishui Lake phages are labelled in red.

Meanwhile, the assignment of DSL-LC02, DSL-LC03, and DSL-LC07 was also evaluated based on nucleotide identity across the genome (genus > 70%; species > 95%) [21]. As shown in Figure 6, Dishui Lake phages of DSL-LC02 (<21.6%), DSL-LC03 (<23.4%), and DSL-LC07 (<37%) had less nucleotide similarity to viruses in the other genera. The similarity between DSL-LC02 and DSL-LC03 was 30.4%, which is higher in comparison to that of the others in *Kyanoviridae* but less than 70%. Clearly, these results confirm that each of these three Dishui Lake phages could be classified to a new genus.

DSL-LC02, DSL-LC03, and DSL-LC07 also shared similarity of the colinear distribution of genes involving the capsid structure, genome package, and DNA replication and transcription with their closest relatives of the same family (Figure 1).

### 3.6. Phylogenetic Analyses

**Figure 7:**
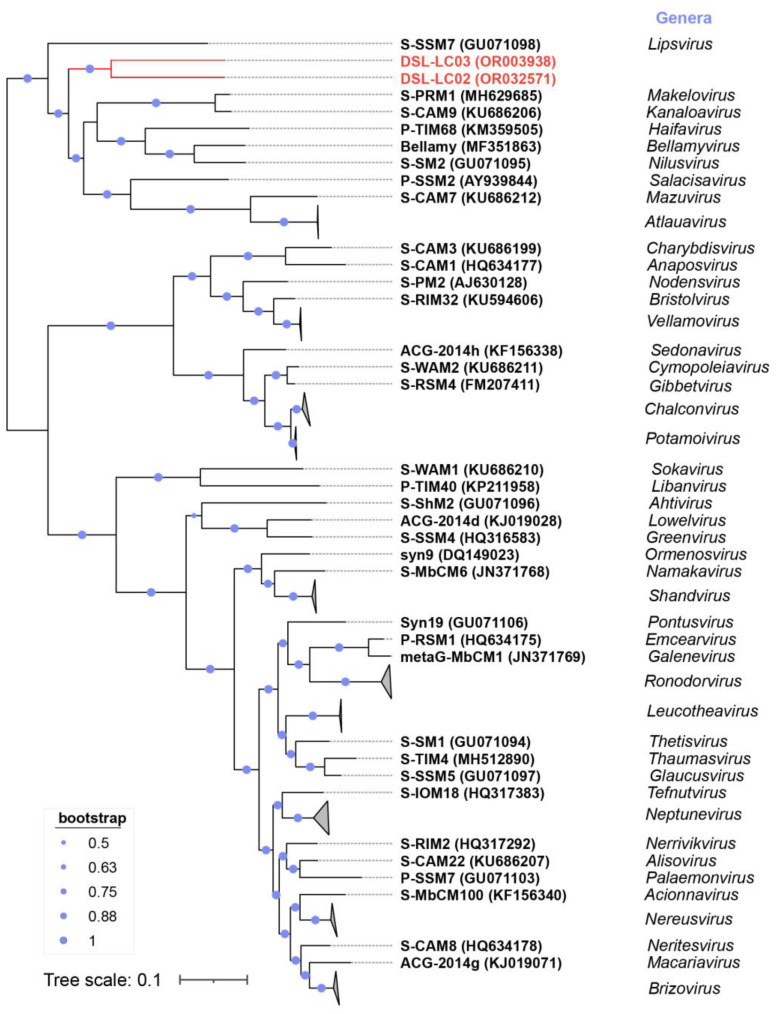
Phylogenetic tree (midpoint rooting) of DSL-LC02, DSL-LC03, and members of *Kyanoviridae* based on 14 core genes concatenated. Viruses in the same genus are collapsed to triangles. Dishui Lake phages are labelled in red.

**Figure 8:**
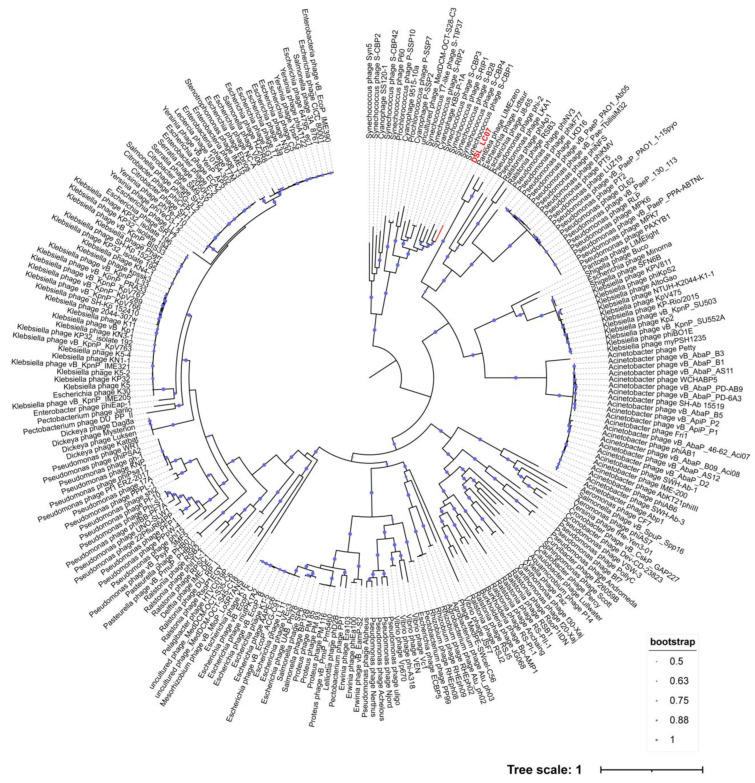
Phylogenetic tree (midpoint rooting) reconstructed by using DNA-dependent RNA polymerases from DSL-LC07 and 244 complete *Autographiviridae* RefSeq genomes in the NCBI virus database. The size of the purple dots represents the bootstrap value. Dishui Lake phages are labelled in red.

Viral hallmark genes refer to relatively conserved genes within a specific group of viruses [59]. The family *Kyanoviridae* contains at least 14 conserved genes of T4-like phages (https://ictv.global/files/proposals/approved) (accessed on 18 April 2023), which were identified in both DSL-LC02 and DSL-LC03 as well (Table S5). These two Dishui Lake phages were clustered together on the tree reconstructed by using the core-gene set and were closely related to the genera of *Makelovirus* and *Kanaloavirus* (Figure 7) like the proteomic tree (Figure 4a). For the *Autographiviridae*, all the members encode a characteristic DNA-dependent RNA polymerase (RNAP) [60], which was also present in DSL-LC07. The RNAP tree showed that DSL-LC07 was grouped together with *Synechococcus* phage S-CPB1, 3 and 4 (*Lirvirus* and *Poseidonvirus*) (Figure 8), which is similar to its placement in the proteomic tree (Figure 4b). 

**Figure 9:**
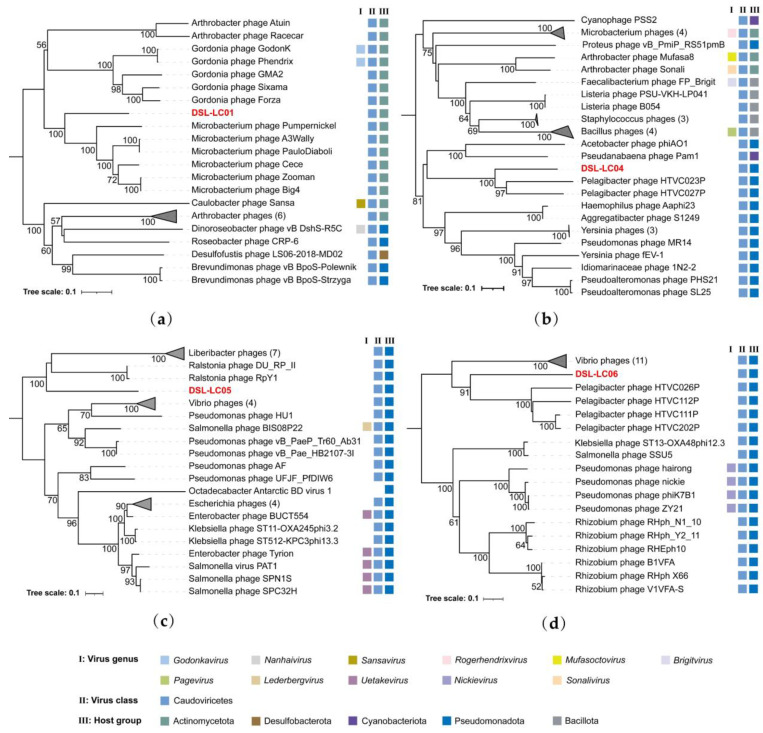
Phylogenetic tree reconstructed by using TerL homologs from DSL-LC01 (**a**), DSL-LC04 (**b**), DSL-LC05 (**c**), DSL-LC06 (**d**), and other viruses. Numbers in the parentheses indicate the number of leaves collapsed. The color diamonds represent the classification information of the viruses and their hosts. Dishui Lake phages are labelled in red.

The *Caudoviricetes* class consists of dsDNA bacteriophages also featuring a conserved gene known as TerL [61]. To further assess the potential relatives of four Dishui Lake phages (DSL-LC01, DSL-LC04, DSL-LC05, and DSL-LC06) without association with known viral families, phylogenetic analyses were carried out using the amino acid sequences of annotated TerL. As shown in Figure 9a, DSL-LC01 TerL was most closely related to that of the *Microbacterium* phages, forming a branch distinct from other ones. The neighboring branch is composed of *Gordonia* phages, with their hosts all belonging to the order *Mycobacteriales* of *Actinomycetes*. TerL of DSL-LC04 and DSL-LC06 were grouped with that of *Pelagibacter* phages that are ubiquitous in marine environments and infect *Pseudomonadota* [62] (Figure 9b,d). DSL-LC05 was clustered with *Ralstonia* and *Liberibacter* phages, with robust bootstrap value support, whose hosts are affiliated with *Pseudomonadota* from freshwater environments (Figure 9c).

### 3.7. Predicted Viral Hosts

The potential hosts of the seven Dishui Lake phages were predicted based on the consistency results of hostG (confidence score > 90) and iPHoP (Table 2). DSL-LC01 seemed to infect the members of *Actinomycetia*. DSL-LC02, DSL-LC03, and DSL-LC07 are likely the viruses of *Cyanobiaceae*, and two of them could be annotated to the level of the host genus. DSL-LC04 and DSL-LC05 may feed on members of *Pseudomonas* and *Enterobacterales*, respectively. DSL-LC06 appeared to prey on members of *Pseudomonadota*. The tRNA genes were identified in DSL-LC01, 02, and 03 (Table 1). The Ala-tRNA (TGC) gene of DSL-LC03 matched to that of *Cyanobacteriota* (Table S6), which is consistent with the hostG and iPHoP results. The other tRNA genes failed to be linked to the potential hosts. No hits were found based on CRISPR spacer-protospacer searches.

## 4. Discussion

The currently studied freshwater viruses have demonstrated that the numerical superiority of the assigned contigs belonged to head-tail bacteriophages of the *Caudoviricetes* [63], but our understanding of freshwater viral diversity and function is far from complete. Assembling complete viral genomes can significantly improve the quality of subsequent systems-wide functional profiling studies, determine all of the genetic components, and better understand how it operates, thereby enabling the discovery of new biological insights [64]. In this study, the majority of viral contigs in Dishui Lake dataset were assigned to unknown virus categories which may play important roles in the lake, and some of them were assigned to the *Kyanoviridae*, containing a large number of cyanoviruses infecting cyanobacteria that frequently dominate in freshwater systems [65]. Importantly, complete genomes of seven Dishui Lake bacteriophages were assembled, analyzed, and taxonomically classified.

Genes that are not involved in viral replication but associated with non-essential viral functions are referred to as AMGs [66], which are hijacked by phages from their hosts, strongly influence microbial metabolism and diversity, and enhance virus fitness by expressing AMGs [67]. DSL-LC01 contains four AMGs (Figure 2). ORF7 encodes D-alanyl-D-alanine carboxypeptidase, which catalyzes distinct carboxypeptidation and transpeptidation reactions during the last stages of cell wall peptidoglycan synthesis, thereby regulating host external structure [68]. ORF125 encodes UDP-galactose mutase (UGM). UGM is an enzyme involved in galactofuranose metabolism and is a critical component in catalyzing the formation of the bacterial cell walls [69]. Thus, DSL-LC01 may inhibit the morphological growth of a host. ORF132 appears to function as DNA methyltransferase that serves to methylate DSL-LC01 genomes, allowing it to evade the host’s restriction enzymes as part of the immune response, and it plays an essential role in gene regulation, potentially relating to virulence [70]. ORF137 protein is homologous to tryptophan 7-halogenase, a halogenating enzyme, which has been recognized as a key factor in incorporating chloride and bromide into activated organic molecules during the biosynthesis of various secondary metabolites [71]. Except for the AMGs, DSL-LC01 also possesses the transcriptional regulator WhiB proteins (ORF69, ORF135), which are exclusively found in actinobacteria and actinobacteriophages and help phages to repress the expression of the host whiB2 gene, thus causing morphological changes and growth inhibition of the host [72]. Notably, over 70% of genes in actinobacteriophages have unknown functions [73], and this is true for DSL-LC01 as well. Collectively, these results suggest that DSL-LC01 is highly likely an actinobacteriophage, encompassing enormous genetic diversity.

Photosynthesis-associated proteins of PsbA and PsbD are only present in the genomes of DSL-LC02, DSL-LC03, and DSL-LC07 (Figure 1). The *psbA* gene is essential as expressed upon phage infection and contributing to maintaining host photosynthesis during infection to increase its fitness [74]. Some lytic phages-encoded glycosyltransferases (GTFs, PF03414.13) (DSL-LC02 ORF132; DSL-LC03 ORF2) glycosylate their DNA to protect it from the host endonuclease restriction system [75]. GDP-L-fucose (DSL-LC02 ORF140; DSL-LC03 ORF209) and GDP-D-mannose 4,6-dehydratase (GMD) (DSL-LC02 ORF142; DSL-LC03 ORF208) participate in the initial step of de novo biosynthesis of GDP-L-fucose which plays a vital role in the bacterial metabolic pathway [76]. They are also present in the cyanophage P-SSM2 infecting *Prochlorococcus*, contributing to its life cycles [77]. Taken together, DSL-LC02, DSL-LC03, and DSL-LC07 are highly likely to be cyanophages, and the presence of these enzymes/proteins constitutes one of the main characteristics of these phages. 

DSL-LC04, DSL-LC05, and DSL-LC06 contain part of the specific functional proteins that have an impact on the host (Figure 2). The 2OG-Fe(II) oxygenase (DSL-LC04 ORF78) plays a pivotal role in a multitude of biological processes, catalyzing the hydroxylation of molecules in microorganisms to regulate lipid metabolism, DNA repair, protein modification, and secondary metabolite production [78]. During phage infection, it regulates host nitrogen metabolism and energy production by modulating intracellular levels of 2-oxoglutarate [79], suggesting the importance and diverse functions of its ability to reprogram host metabolism during infection [80]. Holin (DSL-LC04 ORF72; DSL-LC05 ORF16) generates non-specific “holes” in the host membrane as well as modulates the length of the infective cycle and the release of viable phage particles in the lysis pathway [81]. Glucosamine 6-phosphate N-acetyltransferase (GNA1) (DSL-LC06 ORF40) may be part of a complex glycosylation system, including enzymes involved in nucleotide sugar formation, glycosyltransferases, and glycosidases, independent of their host [82]. Accordingly, the DSL phages may incorporate essential cellular genes from the host to optimize the expression of their own genes while infecting the same hosts [83]. 

The tRNA genes were detected in three of these seven phage genomes (Table 1). It is conjectured that the main difference between bacteriophages with and without tRNA genes lies in the length of their genomes, as bacteriophages containing tRNA genes are significantly longer than those without these genes [84], and virulent phages have more tRNA genes than temperate phages, which may be used in the translation process to synthesize essential proteins for them [83]. Some tRNA genes of DSL-LC02 and DSL-LC03 were matched to those of the *Synechococcus* viruses but not to *Synechococcus*, even though their hosts are very likely to be the *Synechococcus* members. Actually, an average similarity of only 70.7% was shared between the tRNA genes of phages and their hosts in the genus rank [83], and only 7.6% of tRNA genes matched to those of the host on the genus level [85]. This likely results from the mutation rate of tRNA genes, which becomes faster in phages than in hosts due to the high growth rate of phages [86]. Clearly, specific hosts can hardly be predicted with certainty through tRNA gene matching alone.

The dominant bacterial phyla in Dishui Lake correspond to typical freshwater bacterial groups, most of which belong to *Pseudomonadota*, *Actinomycetota*, *Cyanobacteriota*, and *Bacteroidota* [87], consistent with the predicted taxonomic rank of hosts for the Dishui Lake phages (Table 2). Unfortunately, we failed to find the specific cyanobacterium hosts for DSL-LC02, 03, and 07 based on the spacer-protospacer search, even though the CRISPR-Cas systems are predominantly found in the majority of cyanobacteria except for the marine subclade (*Synechococcus* and *Prochlorococcus*) [88]. The occurrence of CRISPR-Cas systems was previously estimated to be in 40% of bacteria and 81% of archaea [89], while recently it has been found that only 10% of bacteria possess such innate immune systems and that many lineages of uncultivated bacteria appeared to almost devoid of CRISPR-Cas systems [90]. Collectively, these findings suggest the challenges remaining in confident prediction of potential hosts of uncultivated viruses through spacer-protospacer matching analysis.

In light of the results of the taxonomic classification (Table S7), four families and three genera were proposed to accommodate these novel uncultivated head-tailed viruses. DSL-LC01, distantly related to other actinobacteriophages, was assigned to the family ‘*Nanparkviridae*’ (a truncation of Nanhuizui Park located in the Dishui Lake sightseeing region). DSL-LC04, DSL-LC05, and DSL-LC06, which infect *Pseudomonadota* members, were classified into the families of ‘*Dishuiviridae*’ (a truncation of Dishui Lake), ‘*Luchaoviridae*’ (Dishui Lake, also known as Luchao Lake), and ‘*Nanhuiviridae*’ (Dishui Lake is located in Nanhui New Town), respectively. Three novel *Synechococcus* phages of DSL-LC02, DSL-LC03, and DSL-LC07 were classified to the genera of ‘*Norislandvirus*’, ‘*Souislandvirus*’, and ‘*Wesilandvirus*’ (North Island, South Island, and West Island; three scenic spots in Dishui Lake), respectively.

## 5. Conclusions

Our study represents the first investigation of bacteriophages in Dishui Lake. The metagenomic datasets have led to the discovery of the complete genomes of seven novel phages. These phages display unique genomic characteristics and could be classified into three new genera within two known families (*Kyanoviridae* and *Autographiviridae*) as well as four new families within *Caudoviricetes*. The potential hosts of these phages belong to the three predominant bacterial phyla (*Cyanobacteriota*, *Pseudomonadota*, and *Actinomycetota*) found in Dishui Lake, with three out of the seven Dishui Lake phages infecting *Synechococcus* of *Cyanobacteriota*. Our findings suggest that there is a vast diversity of viruses yet to be discovered in the lake environment as these phages are only distantly related to known viruses. Additionally, six out of the seven Dishui Lake phages are lytic, which highlights their crucial roles in regulating the predator–prey relationship between hosts and phages. Our study emphasizes the importance of metagenomics in uncovering uncultivated viruses in freshwater environments.

## Figures and Tables

**Table 1:** General features of seven Dishui Lake phages.

Phage	Genome (bp)	%GC	No. of ORF	Annotated ORF	AMGs	tRNA	Lifestyle ^a^
Ρ	129,469	53.6	152	57	4	3	Temperate
DSL-LC02	167,928	36.9	226	102	6	11	Virulent
DSL-LC03	172,805	35.8	210	95	7	11	Virulent
DSL-LC04	61,547	51.3	86	35	0	0	Virulent
DSL-LC05	37,569	56.6	59	31	0	0	Virulent
DSL-LC06	29,811	38.2	44	25	1	0	Virulent
DSL-LC07	45,797	46.1	64	32	1	0	Virulent

^a^ Predicted by BACPHLIP.

**Table 2:** Potential hosts of seven Dishui Lake phages.

Phage	iPhoP	hostG
DSL-LC01	Actinomycetota; Actinomycetia	Actinomycetota; Actinomycetia
DSL-LC02	Cyanobacteriota; Cyanobacteriia; PCC-6307; *Cyanobiaceae*	Cyanobacteriota
DSL-LC03	Cyanobacteriota; Cyanobacteriia; PCC-6307; *Cyanobiaceae*; *Synechococcus*	Cyanobacteriota
DSL-LC04	Pseudomonadota; Gammaproteobacteria	Pseudomonadota; Gammaproteobacteria; Pseudomonadales; *Pseudomonadaceae*; *Pseudomonas*
DSL-LC05	Pseudomonadota; Gammaproteobacteria;	Pseudomonadota; Gammaproteobacteria; Enterobacterales
DSL-LC06	Pseudomonadota	-
DSL-LC07	Cyanobacteriota; Cyanobacteriia; PCC-6307; *Cyanobiaceae*; *Synechococcus*	Cyanobacteriota

## Data Availability

The genomic sequences generated in this study have been deposited in GenBank with the accession numbers of OQ999401 (DSL-LC01), OR032571 (DSL-LC02), OR003938 (DSL-LC03), OR003939 (DSL-LC04), OR003940 (DSL-LC05), OR003941 (DSL-LC06), and OQ999402 (DSL-LC07). Other data generated or analyzed during this study are available from the authors upon request.

## Supplementary Materials

The following supporting information can be downloaded at: www.mdpi.com/article/10.3390/v15102038/s1, Figure S1: Auxiliary metabolic genes identified in Dishui Lake phages; Figure S2: Proteomic subtree of DSL-LC01, 04, 05, and 06; Figure S3: Orthologous gene proportions of Dishui Lake phages of DSL-LC02, 03, and 07; Table S1: 415 viral contigs (>10 kb) primarily identified in the Dishui Lake metagenomic dataset by Cenote-Taker2; Table S2: ORF annotation of Dishui Lake phages; Table S3: 238 relevant viral sequences of Dishui Lake phages retrieved from the IMG/VR4 dataset; Table S4: Classification of Dishui Lake phages based on vConTACT2; Table S5: Fourteen conserved core genes of *Kyanoviridae* detected in DSL-LC02 and 03. Table S6: The tRNA genes of DSL-LC01 and 03 matching to that of bacteria. Table S7: Genomic characteristics and criteria used for the classification of Dishui Lake phages.
