# Supplementary material for: Genomic Analysis and Taxonomic Characterization of Seven Bacteriophage Genomes Metagenomic-Assembled from the Dishui Lake
Source: Viruses. 2023 Sep 30;15(10):2038. doi: 10.3390/v15102038 (PMC10611076; doi:10.3390/v15102038)
Supplement: Supplementary file 1 [file viruses-15-02038-s001.zip › Figures S1-S3.pdf]

viruses

Supplementary Data

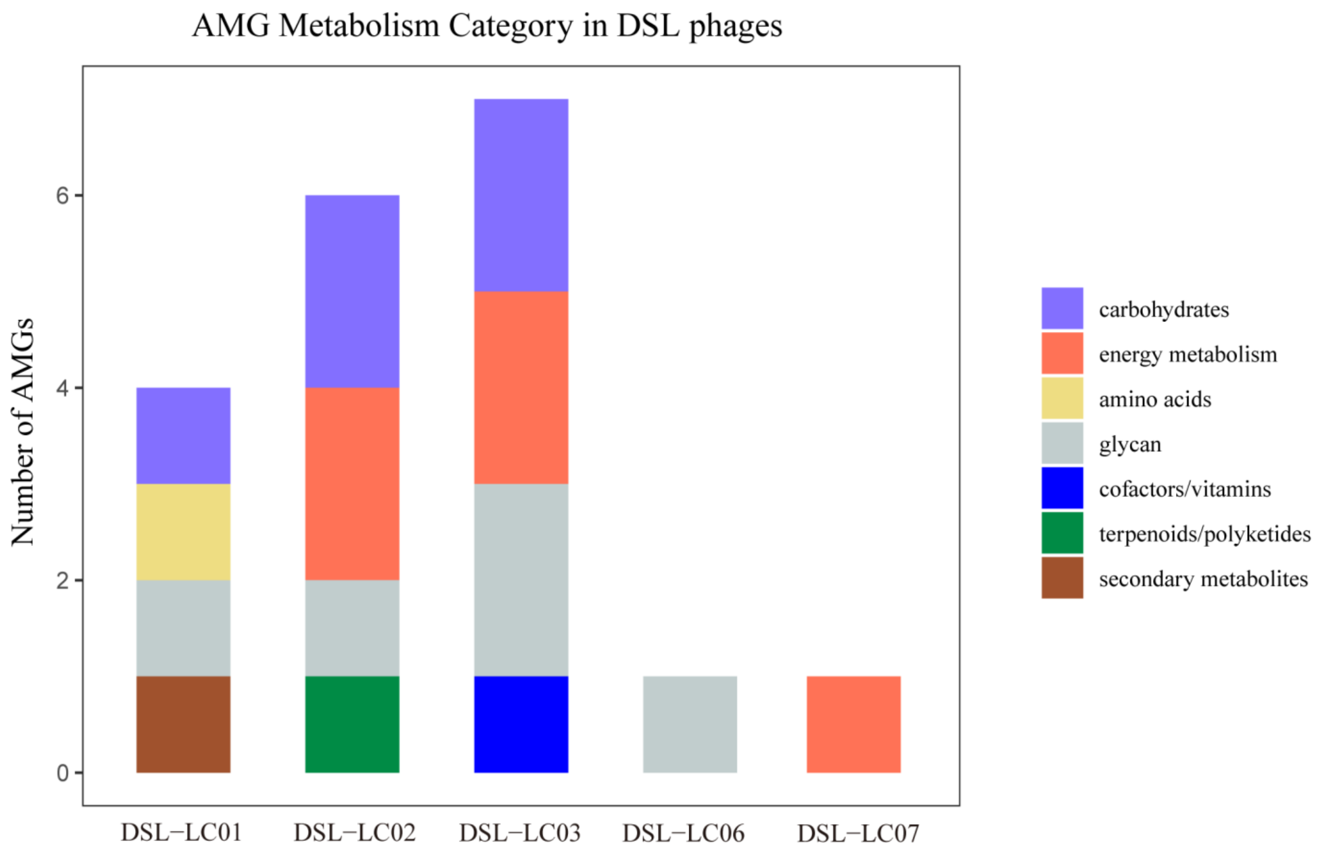

Figure S1. Auxiliary metabolic genes identified in DSL phages.

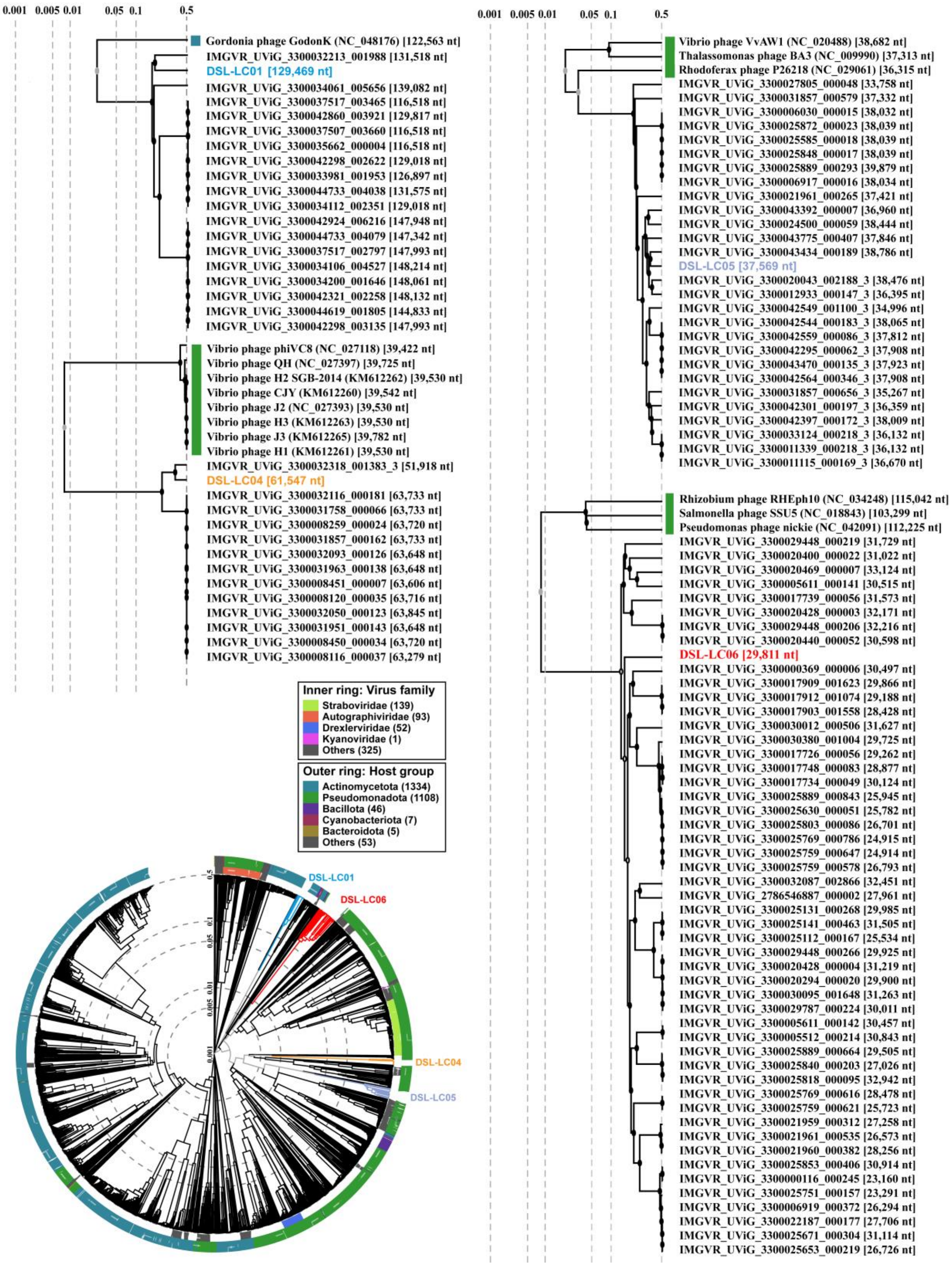

Figure S2. Proteomic subtree of DSL-LC01, 04, 05, and 06. The colorful columns in the subtree indicate host group.

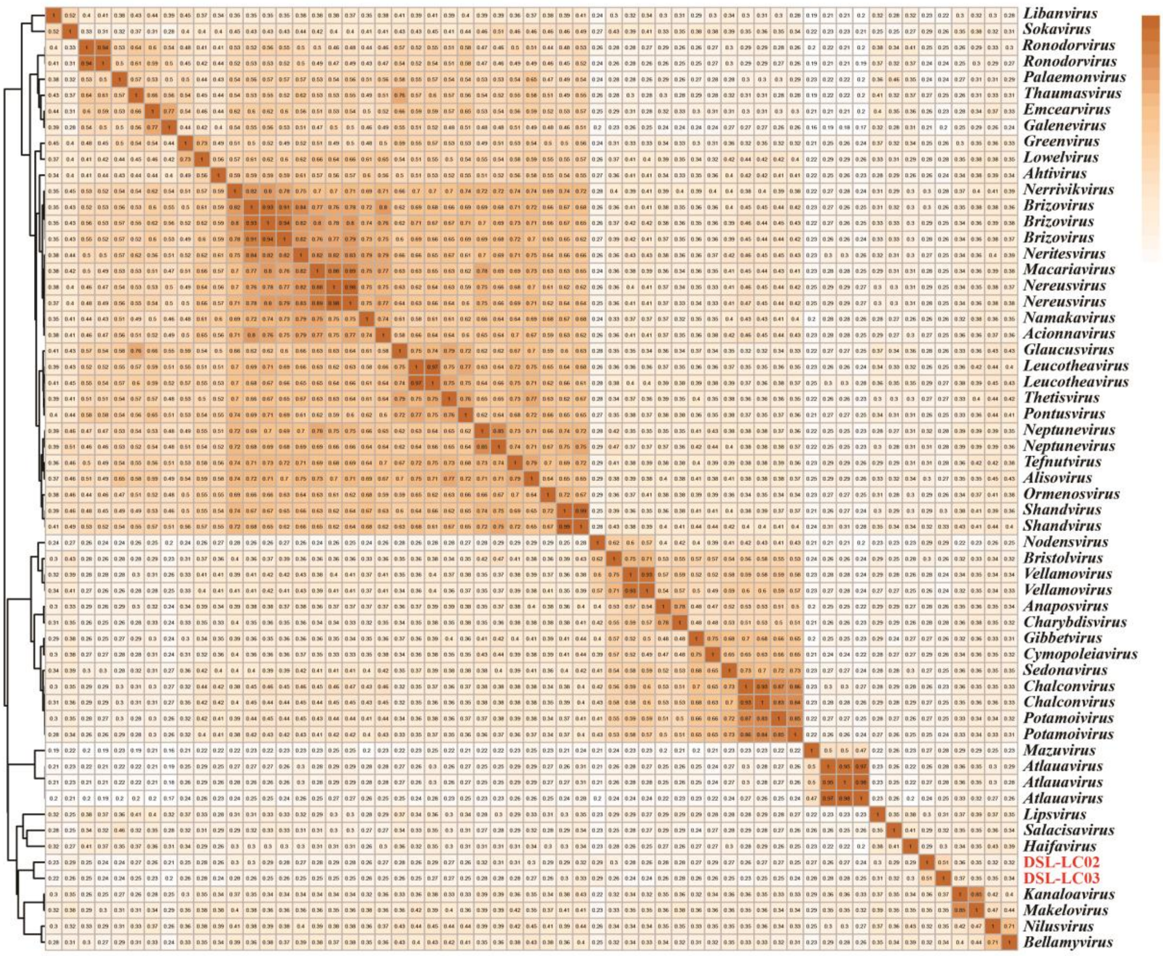

(a)

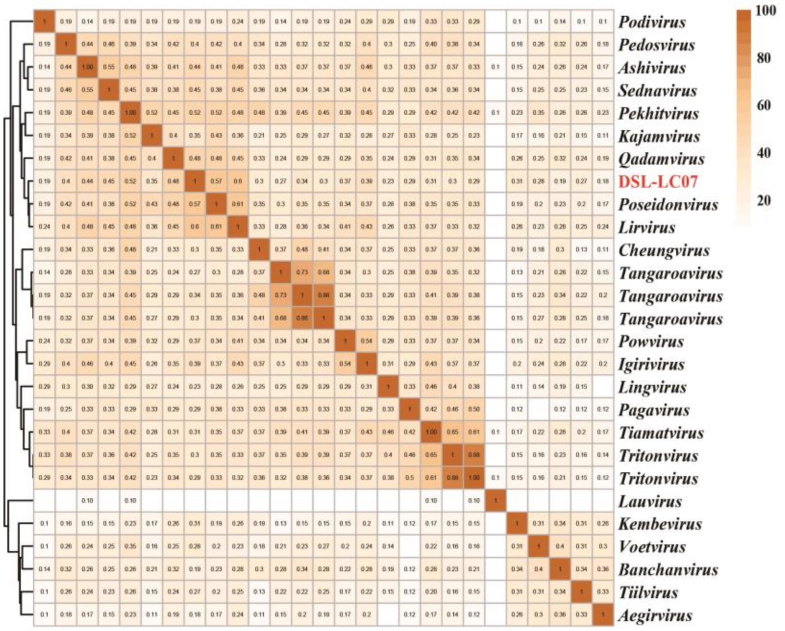

(b)

Figure S3. Orthologous gene proportions of DSL phages of DSL-LC02, 03, and 07. **(a)** The heatmap of DSL-LC02, DSL-LC03, and members of *Kyanoviridae*; **(b)** DSL-LC07 and closely related *Autographiviridae* viruses. Value ≥ 0.1 shown in the boxes.
